# Supplementary material for: Educational Gradients in Drinking Amount and Heavy Episodic Drinking among Working-Age Men and Women in Spain
Source: Int J Environ Res Public Health. 2022 Apr 5;19(7):4371. doi: 10.3390/ijerph19074371 (PMC8998990; doi:10.3390/ijerph19074371)
Supplement: Supplementary file 1 [file ijerph-19-04371-s001.zip › ijerph-1641161-supplementary.pdf]

Table S1. Effective sample of population aged 25–64 years analyzed by sex, age group, period and educational level. Spain, 1997–2017 (No. of people)[a].

	Education	Primary	Secondary 1st stage	Secondary 2nd stage	University	Total
**Sex**	**Period**					
	1997–2007	9205	10841	8200	6575	34821
Men	2009–2017	5763	11509	9281	6216	32769
	1997–2017	14968	22350	17481	12791	67590
	1997–2007	11271	11953	6851	6215	36290
Women	2009–2017	6014	11312	9026	7020	33372
	1997–2007	17285	23265	15877	13235	69662

[a] Sample size refers to the population aged 25–64 years with known values in the variables amount of alcohol intake and the rest of the variables in the table (sex, period and education).

Table S2. General characteristics of the population aged 25–64 by sex and educational level. Spain 1997–2017.

	Sex & education							
	Men				Women			
	Primary	Secondary 1st stage	Secondary 2nd stage	University	Primary	Secondary 1st stage	Secondary 2nd stage	University
**Age (%)**								
25–29	21.4	46.0	50.2	49.0	17.2	41.9	51.7	56.3
30–64	78.6	54.0	49.8	51.0	82.8	58.1	48.3	43.7
**Period (%)**								
1997–2007	57.3	44.0	42.7	47.0	60.9	46.3	39.0	42.7
2009–2017	42.7	56.0	57.3	53.0	39.1	53.7	61.0	57.3
Single	45.3	55.9	55.8	50.8	25.7	39.0	47.2	48.4
Married	48.2	39.6	39.7	44.2	60.6	52.4	45.1	45.3
Separated/divorced	4.3	3.9	4.1	4.4	5.5	6.0	6.3	5.2
Widower	2.3	0.6	0.4	0.6	8.2	2.5	1.3	1.1
**Immigration status (%)**								
Born in Spain	87.2	89.4	86.8	89.1	87.3	88.6	84.4	88.2
Born abroad	12.8	10.6	13.2	10.9	12.7	11.4	15.6	11.8
**Region (%)**								
Andalucía	22.8	18.0	13.9	15.7	23.2	17.8	13.8	14.5
Aragón	2.4	3.0	3.1	3.1	2.4	2.7	2.9	3.2
Asturias	2.1	2.4	2.9	2.6	2.4	2.4	3.0	2.7
Baleares	2.2	2.8	2.3	1.8	1.9	2.7	2.2	2.0
Canarias	5.3	4.8	4.4	3.6	4.8	4.9	4.5	4.0
Cantabria	0.6	1.6	1.6	1.3	0.7	1.6	1.8	1.2
Castilla-La Mancha	5.1	5.2	4.1	3.7	4.6	5.0	3.8	4.2

Castilla y León	6.4	5.6	4.5	4.6	5.4	5.5	4.4	4.9
Cataluña	14.6	15.2	18.0	17.2	14.7	15.1	17.0	17.6
C. Valenciana	10.4	12.8	9.6	9.7	9.6	12.6	10.0	9.1
Extremadura	3.5	2.4	1.8	2.1	3.3	2.7	1.7	1.8
Galicia	7.0	5.8	6.2	5.1	7.0	5.8	5.9	5.9
Madrid	8.4	10.5	15.4	17.7	10.1	11.2	17.8	17.4
Murcia	3.7	2.9	2.3	2.7	3.6	2.8	2.4	1.9
Navarra	1.4	1.7	2.2	2.6	1.5	1.8	2.2	2.6
País Vasco	3.2	4.3	6.5	5.4	3.6	4.5	5.7	6.1
Rioja	0.6	0.7	0.8	0.8	0.7	0.7	0.7	0.7
Ceuta y Melilla	0.3	0.3	0.3	0.3	0.3	0.3	0.3	0.3
**Tobacco smoking in last 30 days (%)**								
Daily	40.5	44.1	36.6	26.2	19.1	33.2	40.0	23.7
Non daily	4.9	5.3	5.9	6.7	3.3	4.3	5.6	6.5
No	54.5	50.6	57.5	67.2	77.6	62.5	63.5	69.8

Table S3. Educational gradient in measures of drinking amount and frequency of heavy episodic drinking in the last 30 days among population and current drinkers aged 25–64. Spain 1997–2017.

	**Men**						Women					
	**Population[a]**			**Current drinkers[b]**			**Population[a]**			**Current drinkers[b]**		
	**PC[c]**	**95%CI**		**PC[c]**	**95%CI**		**PC[c]**	**95%CI**		**PC[c]**	**95%CI**	
**Drinking amount measure**												
Prevalence of 0.1–20 g/day in men and 0.1–10 g/day in women[d]	2.9	2.7	3.1	1.6	1.4	1.7	5.0	4.7	5.3	0.2	0.0	0.3
Prevalence of 21–40 g/day in men and 11–20 g/day in women[d]	−1.1	−1.6	−0.6	−2.3	−2.8	−1.9	3.6	2.8	4.5	−1.0	−1.8	−0.3
Prevalence of HAD[e]	−6.0	−6.7	−5.2	−7.1	−7.8	−6.4	4.7	3.6	5.9	0.1	−1.0	1.1
Mean daily drinking amount[f]	−1.8	−2.1	−1.5	−3.2	−3.5	−2.9	4.7	4.2	5.3	−0.1	−0.5	0.4
**HED measure**												
Prevalence of HED[g]	−0.6	−1.0	−0.1	−1.9	−2.4	−1.5	5.6	4.7	6.5	0.9	0.1	1.7
Prevalence of one HED day[h]	2.3	1.4	3.1	0.8	−0.1	1.6	6.0	4.5	7.4	1.3	−0.1	2.7
Prevalence of 2–3 HED days[h]	0.6	−0.3	1.4	−0.8	−1.7	0.0	5.4	3.7	7.1	0.8	−0.8	2.5
Prevalence of ≥4 HED days[h]	−3.2	−3.8	−2.5	−4.4	−5.1	−3.7	5.3	3.5	7.1	0.4	−1.2	2.1
Mean HED days[i]	−4.0	−4.7	−3.2	−5.2	−6.0	−4.5	4.6	3.1	6.0	−0.2	−1.5	1.1

Legend: **95%CI**: 95% confidence interval. [a] Population aged 25–64 years. [b] People aged 25–64 years with any alcohol intake in the last 30 days. [c] **PC:** Relative percentage change in the corresponding drinking measure per year of education, under the assumption of a linear association, obtained from negative binomial regression model adjusted for simple age, simple calendar-year, region, marital status and immigration status, all entered as dummy variables. [d] Prevalence of the indicated daily amount of alcohol intake in grams among men and women. [e] **HAD**: Heavy average drinking; that is >40 g/day of alcohol intake among men and >20 g/day among

women. [f] Mean daily amount of alcohol intake in grams. [g] Prevalence of any HED episode in the last 30 days. [h] Prevalence of the indicated number of HED days in the last 30 days. [i] Mean number of HED days in the last 30 days.

Table S4. Income inequality in main measures of drinking amount and heavy episodic drinking in the last 30 days among population aged 25–64 by sex, Spain 2007–2017.

Measure of amount of alcohol intake & individual income	Men			Women		
**Drinking prevalence[a]**	*Preval. Ratio[b]*	*95% CI*		*Preval. Ratio[b]*	*95% CI*	
< 10.000€	0.81	0.78	0.84	0.62	0.58	0.65
10.000€-14.999€	0.85	0.82	0.87	0.67	0.64	0.71
15.000€-24.999€	0.93	0.92	0.95	0.83	0.81	0.85
>=25.000€	1.00			1.00		
	Percent change[c]	*95% CI*		*Percent change[c]*	*95% CI*	
Per thousand € of income	0.47	0.41	0.54	1.17	1.07	1.28
**HAD prevalence[d]**	*Preval. Ratio[b]*	*95% CI*		*Preval. Ratio[b]*	*95% CI*	
<10.000€	1.37	1.15	1.63	0.71	0.55	0.92
10.000€-14.999€	1.21	1.02	1.43	0.68	0.54	0.85
15.000€-24.999€	1.01	0.88	1.13	0.79	0.68	0.91
>=25.000€	1.00			1.00		
	Percent change[c]	*95% CI*		*Percent change[c]*	*95% CI*	
Per thousand € of income	−0.63	−1.15	−0.10	1.24	0.72	1.76
**Mean daily drinking amount[e]**	*Mean ratio[b]*	*95% CI*		*Mean ratio[b]*	*95% CI*	
< 10.000€	0.96	0.89	1.04	0.57	0.50	0.64
10.000€-14.999€	0.95	0.89	1.01	0.65	0.59	0.71
15.000€-24.999€	0.97	0.93	1.01	0.78	0.73	0.82
>=25.000€	1.00			1.00		
	Percent change[c]	*95% CI*		*Percent change[c]*	*95% CI*	
Per thousand € of income	0.16	0.00	0.33	1.53	1.29	1.77
**Prevalence of HED [f]**	*Preval. Ratio[b]*	*95% CI*		*Preval. Ratio[b]*	*95% CI*	
< 10.000€	0.81	0.74	0.88	0.57	0.49	0.65
10.000€-14.999€	0.91	0.84	0.98	0.69	0.61	0.79
15.000€-24.999€	0.95	0.88	1.02	0.74	0.66	0.84
>=25.000€	1.00			1.00		
	Percent change[c]	*95% CI*		*Percent change[c]*	*95% CI*	
Per thousand € of income	0.42	0.21	0.63	1.25	0.91	1.60

Prevalence of ≥4 HED days[g]	*Preval. Ratio[b]*	*95% CI*		*Preval. Ratio[b]*	*95% CI*	
< 10.000€	0.97	0.83	1.12	0.46	0.34	0.61
10.000€-14.999€	0.95	0.83	1.10	0.53	0.42	0.69
15.000€-24.999€	1.00	0.87	1.15	0.63	0.50	0.79
>=25.000€	1.00			1.00		
	Percent change[c]	*95% CI*		*Percent change[c]*	*95% CI*	
Per thousand € of income	0.04	−0.42	0.34	1.92	1.28	2.56
**Mean HED days[h]**	*Mean ratio[b]*	*95% CI*		*Mean ratio[b]*	*95% CI*	
<10.000€	0.91	0.76	1.09	0.52	0.41	0.66
10.000€-14.999€	0.86	0.72	1.01	0.60	0.48	0.73
15.000€-24.999€	0.82	0.70	0.96	0.67	0.55	0.82
>=25.000€	1.00			1.00		
	Percent change[c]	95% CI		Percent change[c]	95% CI	
Per thousand € of income	−0.44	−0.84	0.04	1.38	0.83	1.93

Legend: **95%CI**: 95% confidence interval. [a] Prevalence of any alcohol intake. [b] Ratio between the value of the drinking measure at each education level and its value at university level obtained from negative binomial regression adjusted for simple age, simple calendar-year, region, marital status and immigration status, all entered as dummy variables (model I). [c] **PC:** Relative percentage change in drinking measure per thousand € of income under the assumption of a linear association, obtained from model I. [d] Prevalence of heavy average drinking (HAD); that is >40 g/day of alcohol intake among men and >20 g/day among women. [e] Mean daily amount of alcohol intake in grams. [f] Prevalence of any episode of heavy episodic drinking (HED). [g] Prevalence of the indicated number of HED days. [h] Mean number of HED days.

Table S5. Occupational class inequality in main measures of drinking amount and heavy episodic drinking the last 30 days among population aged 25–64 ever employed by sex, Spain 1997–2017.

**Measure of amount of alcohol intake & occupational class**	***Men***			***Women***		
**Drinking prevalence[a]**	*Preval. Ratio[b]*	*95% CI*		*Preval. Ratio[b]*	*95% CI*	
Unskilled manual workers	0.87	0.85	0.90	0.67	0.64	0.71
Skilled manual workers	0.94	0.92	0.96	074	0.72	0.77
Intermediate occupations and non-manual workers	0.96	0.94	0.97	0.88	0.86	0.90
Managers and professionals	1.00			1.00		
**Prevalence of HAD[c]**	*Preval. Ratio[b]*	*95% CI*		*Preval. Ratio[b]*	*95% CI*	
Unskilled manual workers	1.63	1.42	1.87	0.57	0.43	0.76
Skilled manual workers	1.44	1.29	1.61	0.78	0.64	0.95
Intermediate occupations and non-manual workers	1.19	1.09	1.30	0.98	0.87	1.11
Managers and professionals	1.00			1.00		
**Mean daily drinking amount[d]**	*Mean ratio[b]*	*95% CI*		*Mean ratio[b]*	*95% CI*	

Unskilled manual workers	1.12	1.05	1.20	0.57	0.52	0.63
Skilled manual workers	1.12	1.07	1.17	0.74	0.68	0.81
Intermediate occupations and non-manual workers	1.03	0.99	1.06	0.86	0.82	0.91
Managers and professionals	1.00			1.00		
**Prevalence of HED[e]**	*Preval. Ratio[b]*	*95% CI*		*Preval. Ratio[b]*	*95% CI*	
Unskilled manual workers	0.98	0.90	1.06	0.61	0.52	0.73
Skilled manual workers	1.05	0.99	1.11	0.74	0.65	0.85
Intermediate occupations and non-manual workers	1.01	0.96	1.06	0.89	0.82	0.97
Managers and professionals	1.00			1.00		
**Prevalence of ≥4 HED days[f]**	*Preval. Ratio[b]*	*95% CI*		*Preval. Ratio[b]*	*95% CI*	
Unskilled manual workers	1.08	0.84	1.24	0.48	0.34	0.69
Skilled manual workers	1.38	1.25	1.52	0.66	0.51	0.85
Intermediate occupations and non-manual workers	1.05	0.96	1.14	0.84	0.71	0.99
Managers and professionals	1.00			1.00		
**Mean HED days[g]**	*Mean ratio[b]*	*95% CI*		*Mean ratio[b]*	*95% CI*	
Unskilled manual workers	1.25	1.08	1.45	0.60	0.46	0.78
Skilled manual workers	1.32	1.19	1.46	0.81	0.64	1.03
Intermediate occupations and non-manual workers	1.13	1.04	1.23	0.86	0.76	0.98
Managers and professionals	1.00			1.00		

Legend: **95%CI**: 95% confidence interval. [a] Prevalence of any alcohol intake. [b] Ratio between the value of the drinking measure at each education level and its value at university level obtained from negative binomial regression adjusted for simple age, simple calendar-year, region, marital status and immigration status, all entered as dummy variables (model I). [c] Prevalence of heavy average drinking (HAD); that is >40 g/day of alcohol intake among men and >20 g/day among women. [d] Mean daily amount of alcohol intake in grams. [e] Prevalence of any episode of heavy episodic drinking (HED). [f] Prevalence of the indicated number of HED days. [g] Mean number of HED days.

Table S6. Educational gradient in measures of drinking amount and frequency of heavy episodic drinking in the last 30 days among population aged 25–64 ever employed after additionally adjusting for income and occupation. Spain 2007–2017.

	**Men**						Women					
	**Model I[a]**			**Model III[b]**			**Model I[a]**			**Model III[b]**		
**Drinking amount measure**	**PC[c]**	**95%CI**		**PC[c]**	**95%CI**		**PC[c]**	**95%CI**		**PC[c]**	**95%CI**	
Drinking prevalence[d]	1.4	1.2	1.6	1.0	0.8	1.3	3.6	3.3	4.0	2.8	2.4	3.3
Prevalence of HAD[e]	−7.5	−8.7	−7.2	−6.9	−8.5	−5.3	3.7	1.7	5.75	2.8	0.1	5.5
Mean daily drinking amount[f]	−2.1	−2.6	−1.7	−2.4	−3.0	−1.8	3.2	2.5	4.0	2.1	1.1	3.1
**HED measure**												
Prevalence of HED[g]	−1.3	−1.9	−0.7	−1.6	−2.4	−0.9	4.0	2.8	5.1	3.0	1.5	4.5

Prevalence of ≥4 HED days[h]	-4.6	-5.6	-3.5	-4.4	-5.7	-3.1	4.2	1.9	6.5	1.8	-1.2	4.8
Mean HED days[i]	-5.2	-6.3	-4.2	-5.2	-6.5	-3.9	3.3	1.6	5.0	2.0	-0.3	4.3

Legend: **95%CI**: 95% confidence interval. [a] **Model I:** Results obtained from negative binomial regression in the framework of generalised linear models after adjusting for simple age, simple calendar-year, region, marital status and immigration status, all entered as dummy variables. [b] **Model III:** Same as Model I, but additionally adjusting for income level (four categories) and occupational class (four categories). [c] **PC:** Relative percentage change in the corresponding drinking measure per year of education, under the assumption of a linear association. [d] Prevalence of any amount of alcohol. [e] Prevalence of ≥40 g/day of alcohol intake among men and ≥20 g/day among women. [f] Mean daily amount of alcohol intake in grams. [g] Prevalence of any HED episode in the last 30 days. [h] Prevalence of ≥4 HED days in the last 30 days. [i] Mean number of HED days in the last 30 days.
